# Work-related stress and associated factors among health professionals in zone 1, Afar region, Ethiopia

**DOI:** 10.1016/j.heliyon.2022.e12167

**Published:** 2022-12-10

**Authors:** Sadat Mohammed Yesuf, Behailu Tariku Derseh, Daniel Girma, Tadesse Mamo Dejene

**Affiliations:** Department of Public Health, Asrat Woldeyes Health Science Campus, Debre Berhan University, Debre Berhan, Ethiopia

**Keywords:** Work-related stress, Health professionals, Afar region, Ethiopia

## Abstract

**Background:**

In developing nations like Ethiopia, the number of people suffering from work-related stress is rising at an alarming rate, and it is becoming a public health concerns.

**Objectives:**

The goal of this study is to examine work-related stress and associated factors among health care professionals working in governmental and commercial health care facilities in Zone 1 of Ethiopia's Afar region in 2021.

**Methods:**

A comparative cross-sectional survey was done among 435 health professionals working at government and commercial health facilities in Zone 1, Afar, between April 1 and May 30, 2021. Self-administered structured questionnaires were employed to collect data, and multistage sampling was used to reach out to the study participants. To assess occupational stress, the Perceived Stress Scale was employed (PSS-10). To see if there is a difference in stress levels between government and private health practitioners, a chi-square test of independence was used. In multivariable logistic regression, a statistically significant relationship was found with a p-value of less than 0.05.

**Results:**

This study had a total of 435 participants, with a 96.7 percent response rate. Work-related stress was reported by 67.5 percent of government and 47.2 percent of private health professionals, respectively, and overall stress was reported by 57.5 percent. A chi-square test revealed a significant difference in stress between health professionals working in government and private facilities, X2 (1, N = 435) = 18.19, p < 0.001. A monthly income of 4001–5500 ETB, being a male professional, working 40 h per week, having support and assistance at work, job satisfaction, and uncomfortable room temperature were all linked to work-related stress.

**Conclusion:**

Health practitioners in government facilities experienced more stress than those in the private sector. Moreover, the level of work-related stress was high. Effective programs and protocols are needed to maintain a healthy working environment.

## 1 Introduction Background

Work-related stress is something that is created due to the mismatch of people’s ability with their actual knowledge, skills, and coping mechanisms to respond to it [1]. Globally, this problem is estimated to affect one in three employees in general, a public health problem in particular and it affects both developed and developing countries. Moreover, health professionals are the most vulnerable group to stress-related events than other professions [2].

Around 59 million health workers worldwide are exposed to various health risks and hazards as a result of their unsafe working environment. The majority of these problems are caused by psychosocial hazards (shift work, violence, and stress), as most health practitioners are regarded to be healthy persons [3].

In developing countries, the number of people suffering from work-related stress or worsening their condition as a result of stress is increasing at an alarming rate, and it is becoming a public health concern [4]. Even though research in South Africa found that workplace stress among doctors was 51 percent [5], there is limited evidence in Africa about the incidence of work-related stress. Work-related stress was given far less attention in Ethiopia; yet, certain research found a significant frequency of work-related stress among health professionals [2, 6, 7]. Although there is little research on work-related stress in Ethiopian public health facilities, the amount of work-related stress and its key drivers among Ethiopian health workers in the Afar region have not been investigated. Furthermore, little research has been done to compare the levels of work-related stress experienced by health professionals working in public and private health facilities. As a result, this study is expected to fill these gaps by addressing the magnitude of work-related stress, comparing stress levels between government and private health facilities, and identifying contributing factors for stress among health professionals, which is important for health professionals, patients, the health facility, and stakeholders.

## 2 Methods

### 2.1 Study design and setting

A comparative cross-sectional study was conducted among health professionals who work at both governmental and private health institutions found in Zone one (Awsi Rasu), afar region, from April 1 -May 30, 2021. Afar Region has an estimated population of 1.9 million people. Awsi Rasu is one of five zones found in the Afar region of Ethiopia, which is located 591 km far from the capital city of Ethiopia, Addis Ababa. From government institutions, there are 1 general hospital, 2 primary hospitals, 26 public health centers, 94 health posts, and from private facilities there are 5 primary clinics, 32 medium clinics, 2 specialty clinics, and 41 pharmacies, which are found within the zone. There are 1305 health professionals of which 907 of health professionals was from the government and 398 health professionals were from private health facilities.

### 2.2 Populations

The study's source population was health professionals in the Afar region. Health care professionals working in both private and public health facilities (hospitals, health centers, and clinics) in Zone 1, Afar region, who met the qualifying criteria, were included in this study as the study population. The criteria for participation were health care personnel who have worked for at least 6 months in both private and public health facilities (hospitals, health centers, and clinics).

### 2.3 Sample size and sampling technique

Two population proportion formula was used to calculate the sample size. The previous study assessed the prevalence rate of job-related stress to be 68.2 percent for public health professionals and 50 percent for private health professionals (8). Using the assumptions of (critical value of the normal distribution at α/2 = 1.96), Zβ (critical value of the normal distribution at β = 0.842), n1 = n2 (Sample size from governmental and private health facilities), the calculated sample size was 214 (107 of each). Finally, by adding 5% for the non-response rate (if a health care provider refuses to complete and return a filled questionnaire) and multiplying the sample size by two (2) for the design effect, the total sample size is 450.

**Figure 1:**
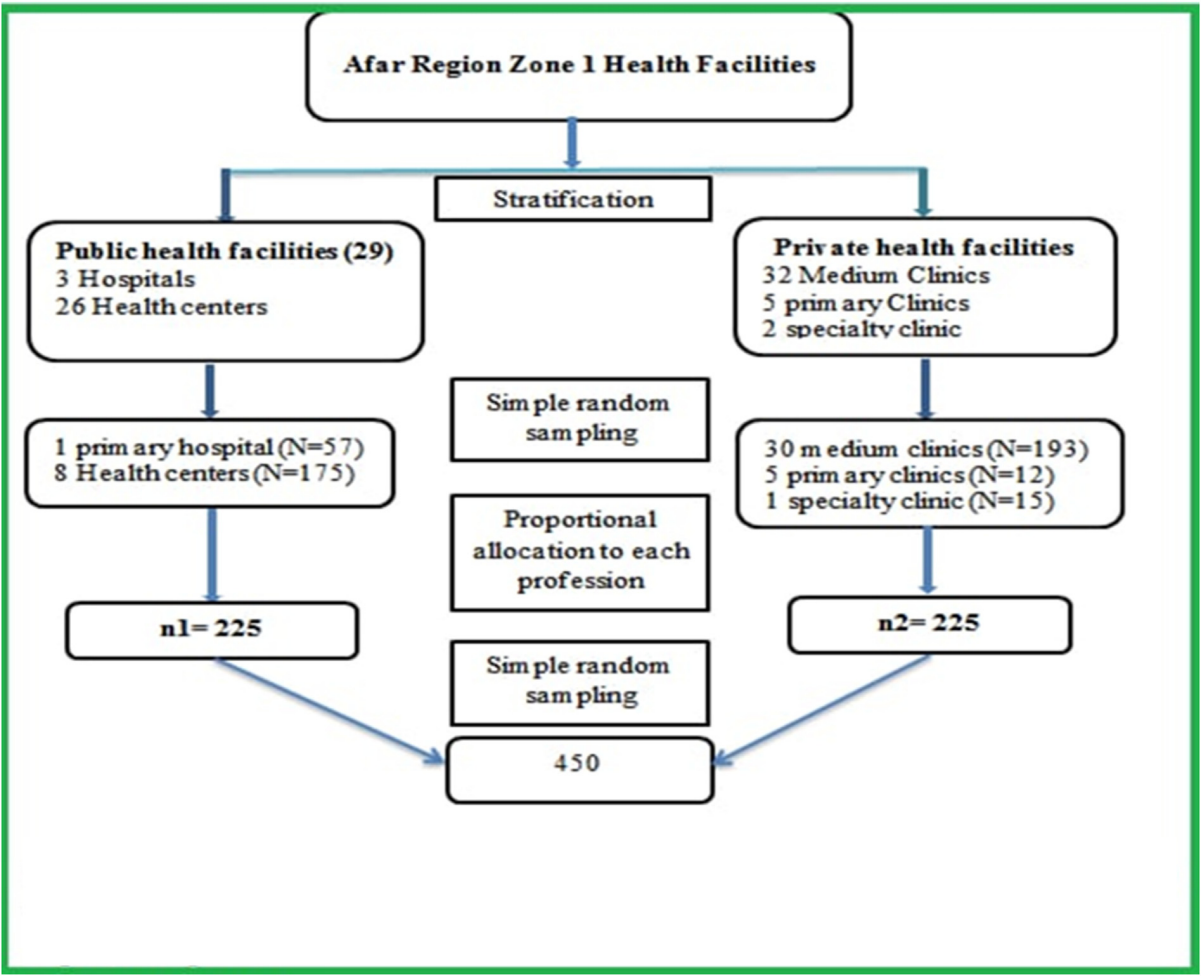
Schematic Presentation of the Sampling Procedure for the study of work related stress among health professionals of working on public and private health facilities of Zone 1, Afar region, Ethiopia 2021.

The researchers employed a multi-stage sampling technique. The health facilities were first classified as public or private, and then a lottery was used to select one primary hospital, eight health centers, and 36 clinics from each facility. After then, the proportional allocation was used to deal with the sample size. Finally, a simple random selection approach was used to pick the participants (Figure 1).

### 2.4 Data collection tools and procedures

A structured, self-administered questionnaire was used to collect data. The questionnaire is divided into four sections: the first section contains socio-demographic data derived from the Ethiopian demographic health survey (EDHS), the second section contains workplace factors, and the third section contains the job satisfaction measurement instrument Satisfaction of Employees in Health Care (SEHC), and the fourth section contains the Perceived Stress Scale-10 (PSS-10). The Perceived Stress Scale (PSS) is a validated and reliable internationally accepted instrument that can be applied to a variety of situations [8]. SEHC is a survey instrument that has been used to assess staff satisfaction in low-income nations like Ethiopia [9]. To maintain consistency, the data collection tool was first translated into Amharic and then back into English as a quality control measure. In addition, the questionnaire was pre-tested on 5% of the sample of a similar population in a different zone (Zone 3). Based on the results of the pretest, some small changes to the questionnaire were made, as well as a redesign of the interview process. Finally, Cronbach alpha (Cronbach alpha ≥0.7) was used to verify the tool's validity and reliability.

### 2.5 Operational definition

**Work-related stress** - Described as professionals who scored above the mean perceived stress scale to have work-related stress and score below the mean perceived stress scale shows no work-related stress [8].

**Perceived stress scale (PSS-10)** - Assesses perceived stressful experiences or stress responses over the previous month with a 5-point Likert scale (0 = never and 4 = very often), which comprised of 10 items with a possible range of scores from 0 to 40. For each sub-scale of WRS, the participant’s responses to each item score were considered [8].

**Job satisfaction** - Health professionals’ positive perceived emotion on the appraisal of their jobs, respondents with an average score of less than the mean value were classified as dissatisfied with the SEHC scale, and those with mean scores and above were considered as satisfied [9].

**Satisfaction of employees in health care (SEHC)** - measures job satisfaction with a possible range of scores from 1 to 72. Overall job satisfaction was measured on a four-point Likert scale with the value, ranging from 1 (strongly disagree) to 4 (strongly agree) [9].

### 2.6 Data processing and analysis

Data were entered using Epi-data version 3.1, and exported to SPSS version 22, to accomplish further data exploration procedures; along with the required statistical data analysis methods. The outcome variable was dichotomized into two groups (1 = stress and 0 = no stress). Descriptive statistics were used to summarize the result and reported using frequencies and percentages. Association between outcome and independent variables tested on bi-variable logistic regression. Variables with P-value ≤0.2 were entered into the final regression model. A multicollinearity test was performed using collinearity diagnostics and has shown the absence of multicollinearity (since the VIF <10). Moreover, the model adequacy was checked using the Hosmer-Lemeshow goodness of fit statistical method; and the result revealed its fitness to use it for further interpretation. Finally, a P-value of less than 0.05 in the final model was considered as the level of statistical significance and reported along with the adjusted odds ratio and 95% CI.

### 2.7 Ethical consideration

The Ethical Review Committee (ERC) of Debre Berhan University's Asrat Woldeyes Health Sciences Campus gave their approval (Protocol no. 53/21/CHS/SPH; date: 16/04/2021). Moreover, a formal letter of permission was obtained from selected private and public health facilities. Before the actual data collection, informed verbal consent was obtained from the respondents as stated in the ethical clearance letter. Privacy and confidentiality of the data were assured making the questionnaire anonymously.

## 3 Results

A total of 435 study participants were interviewed, giving a total response rate of 96.7% (435/450).

### 3.1 Socio-demographic characteristics

Males made up the bulk of the 257 responders (59.1%). Nurses made up 48.8% of the total study participants in government and 36 percent in private health institutions, public health officers made up 7.7% of the total study participants in government and 11.7 percent in private health institutions, and general practitioners (doctors) made up 2.7 percent and 9.4% of the total study participants in government and private health institutions, respectively (Table 1).

**Table 1:** Socio-demographic characteristics of health professionals working in zone 1, Afar region 2021.

Variables		Type of institutions	
		Governmental (%)	Private (%)
Age	<25	66(29.8%)	44(20.5%)
	25–29	93(42.1%)	103(48%)
	>29	62(28%)	67(31.4%)
Sex	Male	129(58.4%)	128(59.8%)
	Female	92(41.6%)	86(41.2%)
Marital status	Single	114(51.6%)	98(45.8%)
	Married	91(41.2%)	102(47.7%)
	Divorced/widowed/separated	16(7.2%)	14(6.5%)
Monthly income	<4000	19(8.6%)	0
	4001–5500	94(42.5%)	68(31.8%)
	5501–7000	62(28%)	67(31.3%)
	>7000	46(20.8%)	79(37%)
Health professionals	Medical doctor	6(2.7%)	20(9.3%)
	Pharmacy	25(11.3%)	28(13.1%)
	Public health	17(7.7%)	25(11.7%)
	Nurse	108(48.9%)	78(36.4%)
	Midwifery	34(15.4%)	17(8%)
	Laboratory	24(10.8%)	34(16%)
	**Others**∗∗	7(3.16%)	12(5.6%)
Educational level	Diploma	132(59.7%)	114(53.3%)
	Degree and above	89(40.3%)	100(46.7%)
Professional experience	<2 years	95(43%)	66(30.8%)
	2–5 years	84(38%)	94(44%)
	>5 years	42(19%)	54(25.2%)

**Others**∗∗ (Emergency Surgeon, Gynecologist, Internist, Obstetrician, Pediatrician, Optometry, Radiology and Anesthesia).

### 3.2 Working environment conditions

Out of the total study participants, 200 (46%) worked in the outpatient department, and the majority (317, or 73%) were assigned to night/weekend duty in addition to their normal work shift (84.6 percent of governmental and 60.7 percent of private health professionals). In terms of workload, around 145 health professionals from government health institutions worked approximately 40 h per week, while approximately 44 percent of health professionals from private facilities worked more than 50 h per week (Table 2)**.**

**Table 2:** Working environment conditions of health professionals in zone 1, Afar region, Ethiopia, 2021.

Working environment conditions		Type of institutions		Total N (%)
		Governmental N (%)	Private N (%)	
Workload	40 h/week	111(50.2%)	34(15.9%)	145(33.3%)
	41–50 h/week	67(30.3%)	86(40.2%)	153(35.2%)
	>50 h/week	43(19.5%)	94(44%)	107(24.5%)
Assigned to night/weekend duty	All the time	18(8.1%)	27(12.6%)	45(10.3%)
	Sometimes	169(76.5%)	103(48.1%)	272(62.5%)
	Not at all	34(15.4%)	84(39.3%)	118(28.2%)
Currently working department	Emergency	26(11.8%)	18(8.4%)	44(10.1%)
	Inpatient/ward	15(6.8%)	11(5%)	26(6%)
	MCH	36(16.3%)	18(8.4%)	54(12.4%)
	OPD/radiology	94(42.5%)	106(49.5%)	200(46%)
	Pharmacy/laboratory	50(22.6%)	61(28.5%)	111(25.5%)
Availability of enough staffs	Yes	147(66.5%)	160(74.8%)	307(70.6%)
	No	74(33.5)	54(25.2%)	128(29.4%)
Receiving necessary equipment and supplies	Often	154(69.7)	175(81.8%)	329(75.6%)
	Sometimes	67(30.3%)	39(18.2%)	106(24.4%)
Injured while at work during last 12 months	Yes	11(5%)	8(3.7%)	19(4.4%)
	No	210(95%)	206(96.3%)	416(95.6%)
temperature uncomfortable	Yes	152(67.8%)	160(74.8%)	312(71.7%)
	No	69(31.2%)	54(25.2%)	123(28.3%)

### 3.3 Interpersonal relationship and career development characteristics

Among the total participants, 86% of governmental and 91% of private professionals responded that they were getting enough support and assistance in their workplace, and also 17 and 9 of governmental and private professionals respectively were involved in interpersonal conflict at their workplace (Table 3).

**Table 3:** Interpersonal relationship and career development characteristics of health professionals working in governmental and private facilities of zone 1, Afar region, Ethiopia 2021.

Variables		Type of institutions		Total (%)
		Governmental N (%)	Private N (%)	
Do Experience violence from patients or members of the community	Yes	57(25.8%)	55(25.7%)	112(25.7%)
	No	164(74.2%)	159(74.3%)	323(74.3%)
Do you feel getting enough support and assistance	Yes	190(86%)	195(91.1%)	385(88.5%)
	No	31(14%)	19(8.9%)	50(11.5%)
Do you currently involve in any conflict at your workplace?	Yes	204(92.3%)	205(96%)	409(94%)
	No	17(7.7%)	9(4%)	26(6%)
Fair and regular career development opportunities	Yes	157(71%)	124(58%)	281(64.6%)
	No	64(29%)	90(42%)	154(35.4)
Job trainings	often	31(14%)	32(15%)	63(14.5%)
	sometimes	64(29%)	83(38.8%)	147(33.8%)
	Rarely/not at all	126(57%)	99(46.2%)	225(51.7%)
Job satisfaction	Satisfied	113(51.1%)	133(62.1%)	246(56.5%)
	Dissatisfied	108(48.9%)	81(37.9%)	189(43.5%)
Plan to change the current work in the coming years	Yes	77(34.8%)	66(30.8%)	143(32.9%)
	No	69(31.2%)	91(42.5%)	160(36.8%)
	I don’t know	75(34%)	57(26.7%)	132(30.3%)

### 3.4 Prevalence of work-related stress

**Figure 2:**
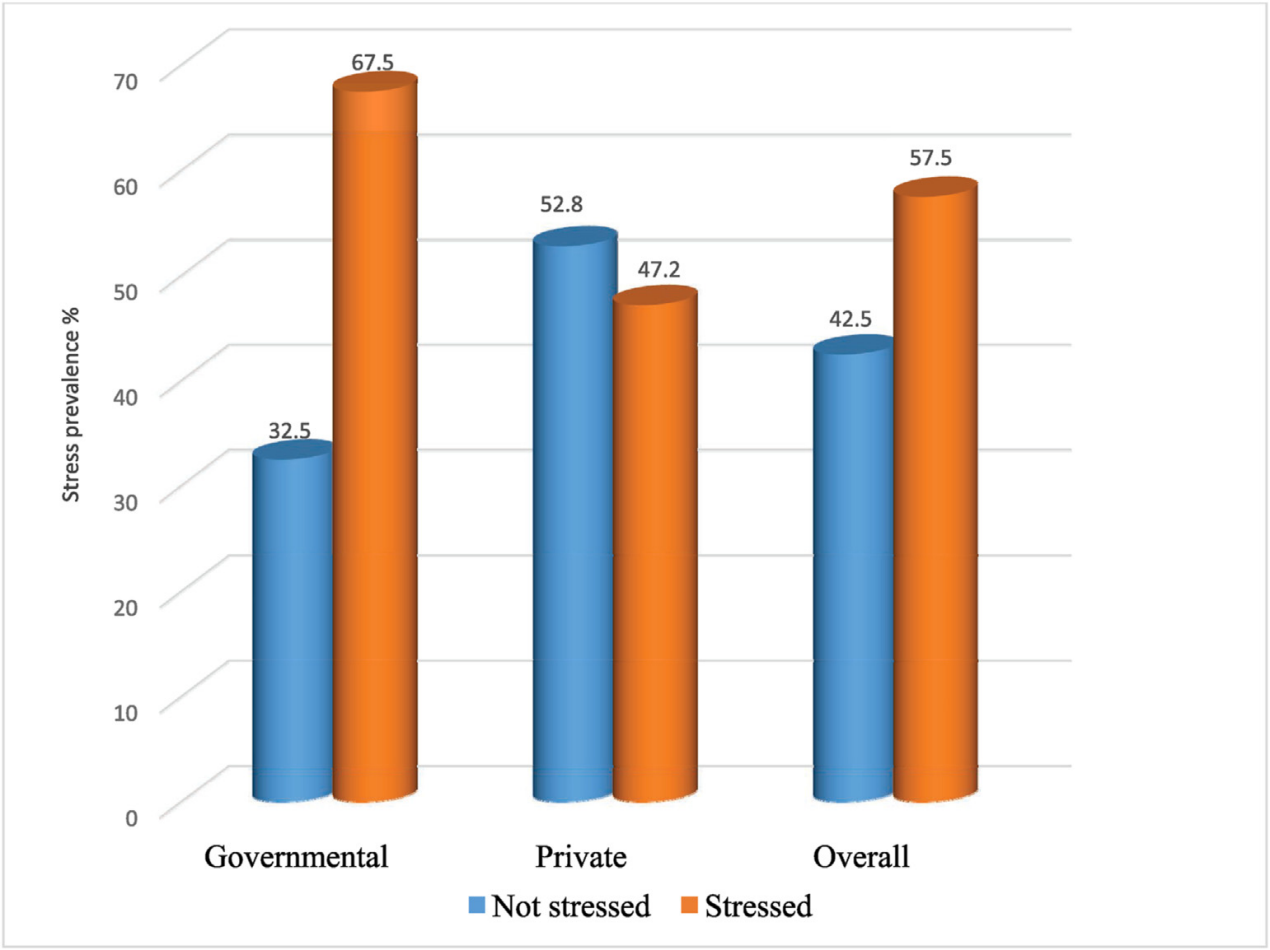
Prevalence of work-related stress among health professionals working in public and private health facilities of Zone 1, Afar region, Ethiopia, 2021.

The overall prevalence of work-related stress was 250 (57.5 percent), 95 percent CI (52.8, 62.1), and the prevalence among governmental facilities health professionals was 149 (67.5 percent), 95 percent CI (61.2, 73.6), while the prevalence among private facilities health professionals was 101 (47.2 percent), 95 percent CI (40.5, 53.9). To see if there is a difference in stress levels between government and private health practitioners, a chi-square test of independence was used. A chi-square test revealed a significant difference in stress between health professionals working in government and private facilities, X^2^ (1, N = 435) = 18.19, p < 0.001. Health practitioners in government facilities experienced more stress than those in the private sector (Figure 2).

### 3.5 Determinants of work-related stress

Age, sex, marital status, educational status, the health professional's background, and monthly wage were among the socio-demographic parameters included for the final model. According to the findings, the study participants' monthly income was substantially linked to work-related stress. As a result, in the overall analysis, health professionals with lower monthly incomes were more likely to be exposed to WRS. When compared to those who earn more than 7000 Ethiopian birrs, the likelihood of work-related stress among health professionals earning 4001–5500 Ethiopian birrs was approximately three times greater [AOR = 2.83; 95 percent CI (1.2–6.60)] (Table 4)**.**

**Table 4:** Socio-Demographic Factors and its association with work-related stress among health professionals, zone 1 Afar region, Ethiopia 2021.

Socio-demographic variables		Work-related stress		COR (95%CI)	AOR (95%CI)
		Yes	No		
Institution	Government	149	72	**2.3(1.6–3.41)**	**3.8(2.03–7.1)**
	Private	101	113	1	1
Age	<25	51	59	0.73(0.44–1.21)	0.56(0.26–1.2)
	25–29	129	67	1.6(1.03–2.55)	2.0(0.97–3.7)
	>29	70	59	1	1
Sex	Male	131	126	0.52(0.35–0.76)	0.7(0.38–1.26)
	Female	119	59	1	1
Marital status	Single	105	107	1.14(0.53–2.4)	1.2(0.3–3.7)
	Married	130	63	2.3(1.1–5.1)	3.6(0.99–10.3)
	Divorced/widowed/separated	14	16	1	1
Monthly income	<4000	16	3	6.4(1.77–22.9)	5.3(0.95–29.2)
	4001–5500	110	52	2.5(1.6–4.0)	**2.83(1.2-6.6)∗**
	5501–7000	67	62	1.3(0.8–2.11)	0.95(0.43–2.1)
	>7000	57	68	1	1
Educational level	Diploma	152	94	1.5(1.02–2.2)	1.03(0.6–1.95)
	Degree and above	98	91	1	1
Professional experience	<2 years	103	58	1.63(0.98–2.6)	1.9(0.83–4.2)
	2–5 years	97	81	1,1(0.67–1.81)	0.9(0.44–1.87)
	>5 years	50	46	1	1

Workload, job satisfaction, and an uncomfortable room temperature, on the other hand, were all linked to work-related stress. Health professionals who worked 40 h per week had a 70% lower risk of work-related stress than those who worked more than 50 h per week [AOR = 0.30; 95 percent CI (0.14–0.68)].

Health professionals who work under Uncomfortable room temperature were more than 4 times more likely to be stressed [AOR = 4.30; 95% CI (2.24–8.10)] than the referent (Table 5).

**Table 5:** Work-related conditions and their association with work-related stress among health professionals, zone 1 Afar region, Ethiopia 2021.

Work-related conditions		Work-related stress		COR (95%CI)	AOR (95%CI)
		Yes	No		
Workload	40 h/week	72	73	0.44(0.27–0.7)	**0.3(0.14-0.68) ∗**
	41–50 h/week	83	70	0.52(0.32–0.85)	0.5(0.25–1.02)
	>50 h/week	95	42	1	1
Assigned to night/weekend duty	All the time	33	11	3.4(1.6–7.2)	3.31(0.97–9.34)
	Sometimes	161	111	1,6(1.02–2.51)	1.3(0.69–2.4)
	Not at all	56	63	1	1
Currently working department	Emergency	34	10	3.72(2.7–8.2)	4.4(0.97–16.4)
	Inpatient/ward	16	10	1.75(0.73–4.1)	2.27(0.5–10.05)
	MCH	28	26	1.18(0.62–2.2)	1.01(0,25–4.06)
	OPD/radiology	119	81	1.6(1.01–2.5)	1.98(0.64–6.1)
	Pharmacy/laboratory	53	58	1	1
Is there enough staff	Yes	169	138	0.71(0.47–1.0)	0.78(0.4–1.52)
	No	81	47	1	1
Having clear responsibility	Yes	210	163	0.7(0.4–1.2)	1.2(0.61–2.34)
	No	40	22	1	
Availability of equipment and supplies	Often	185	144	0.8(0.52–1.27)	0.89(0.51–1.57)
	Sometimes	65	41	1	1
Injured while at work during last 12 months	Yes	13	6	1.63(0.61–4.39)	2.2(0.7–6.58)
	No	237	179	1	1
Uncomfortable room temperature	Yes	208	104	3.86(2.52–5.9)	**4.3(2.24-8.1) ∗**
	No	42	81	1	1

Moreover, satisfied health professionals with their jobs [AOR = 0.35; 95% CI (0.22–0.61)] were 65 % less likely to have work-related stress than those unsatisfied health professionals (Table 6).

**Table 6:** Interpersonal relationship and career development factors of work-related stress among health professionals, zone 1 Afar region, Ethiopia 2021.

Interpersonal relationship and career development factors		Work-related stress		COR (95%CI)	AOR (95%CI)
		Yes	No		
Having support and assistance from staffs	Yes	210	175	0.3(0.2–0.71)	0.35(0.18–1.04)
	No	40	10	1	1
Receiving necessary trainings	Often	31	32	0.48(0.3–0.85)	0.54(0.23–1.26)
	Sometimes	69	78	0.44(0.29–0.67)	0.4(0.21–1.07)
	Rarely/not at all	150	75	1	1
involved in any conflict at your workplace	Yes	19	7	2.09(0.86–5)	3.75(0.98–11.8)
	No	231	178	1	1
Experience violence from patients or members of the community	Yes	74	38	1.6(0.94–2.54)	1.24(0.61–2.5)
	No	176	144	1	1
Having career development opportunities	Yes	162	119	1.02(0.69–1.52)	0.89(0.56–1.41)
	No	88	66	1	
Job satisfaction	Satisfied	112	134	0.31(0.2–0.46)	0.35(0.22–0.61) ∗
	Dissatisfied	138	51	1	1
Plan to change the current workplace in the coming years	Yes	97	46	1.32(0.81–2.18)	0.7(0.35–1.42)
	No	72	88	0.51(0.32–0.82)	0.66(0.34–1.2)
	I don’t know	81	51	1	1

### 3.6 Determinants of work-related stress by governmental - private category

In governmental health facilities, the sex of the respondents was found to be significantly associated with work-related stress, where being male has a 66 % [(AOR = 0.34; 95% CI (0.15–0.78)] less chance to have work-related stress than female professionals (Table 7). Similarly, workload, job satisfaction, and uncomfortable room temperature were important significant factors of work-related stress in both governmental and private institution health professionals (Table 8)**.**

**Table 7:** Socio-Demographic Factors and its association with work-related stress among health professionals by health institution category, zone 1 Afar region, Ethiopia 2021.

Socio-demographic factors		Governmental			Private		
		Stress	No stress	Adjusted OR (95% CI)	Stress	No stress	Adjusted OR (95% CI)
Age	<25	35	31	0.16(0.05–0.5)	16	28	0.88(0.3–2.51)
	25–29	72	21	3.0(0.98–8.2)	57	46	1.62(0.76–3.4)
	>29	42	20	1	28	39	1
Sex	Male	79	50	**0.34(0.15-0.78) ∗**	52	76	**0.62(0.32–1.2)**
	Female	70	22	1	49	37	1
Marital status	Single	76	38	0.73(0.19–2.8)	30	68	0.85(0.22–3.3)
	Married	65	26	1.4(0.34–5.5)	65	37	2.6(0.7–9.73)
	Divorced/widowed/separated	8	8	1	6	8	1
Monthly income	<4000	16	3	1.5(0.5–10.8)	0	0	-
	4001–5500	72	22	1.49(0.4–5.14)	38	30	2.0(0.69–5.86)
	5501–7000	36	26	0.4(0.13–1.35)	31	36	1.3(0.48–3.52)
	>7000	25	21	1	32	47	1
Profession	Medical doctor	3	3	0.43(0.02–8.4)	9	11	2.4(0.4–17.0)
	Laboratory	20	4	6.5(0,99–32.06)	18	16	1.97(0.6–6.94)
	Public health	13	4	1.53(0.16–14.9)	15	10	3.03(0.5–18.0)
	Nurse	81	27	1.7(0,23–12.5)	40	38	1.4(0.3–6.92)
	Midwifery	15	19	0.53(0.08–3.6)	7	10	1.23(0.18–5.7)
	**Others∗**	2	5	0.2(0.01–2.3)	4	8	0.74(0.12–5.2)
	Pharmacy	17	17	1	8	20	1
Education	Diploma	93	39	0.97(0.4–2.63)	59	55	1.48(0.69–1.3)
	Degree	56	33	1	42	58	1
Experience	<2 years	71	24	6.4(0.98–23.2)	32	34	0.8(0.29–2.19)
	2–5 years	53	31	2.1(0.7–6.53)	44	50	0.71(0.3–2.2)
	>5 years	25	17	1	25	29	1

Others∗: Emergency surgeon/gynecologist/internist/obstetrician/pediatrician/optometry/anesthetists.

**Table 8:** Working conditions and their association with work-related stress among health professionals by institutional category, zone 1 Afar region, Ethiopia 2021.

Working conditions		Governmental			Private		
		stress	No stress	Adjusted OR (95%CI)	stress	No stress	Adjusted OR (95%CI)
Workload	40 h/week	62	49	**0.08(0.02-0.3) ∗**	10	24	**0.32(0.12-0.85) ∗**
	41–50 h/week	49	18	**0.24(0.06-0.9) ∗**	34	52	0.46(0.33–1.0)
	>50 h/week	38	5	1	57	37	1
Assigned to night/weekend duty	All the time	15	3	4.6(0.8–24.03)	18	8	3.2(0.99–9.5)
	Sometime	117	52	2.84(0.98–8.03)	44	59	0.73(0.36–1.46)
	Not at all	17	17	1	39	46	1
Currently working department	Emergency	23	3	11.2(0.94–24.8)	11	7	1.9(0.43–8.44)
	Inpatient/ward	9	6	4.4(0.52–26.7)	7	4	2.5(0.33–17.5)
	MCH	22	14	2.3(0.4–13.3)	6	12	0.49(0.12–4.63)
	OPD/radiology	66	28	3,53(0.6–21.2)	53	53	1.9(0.5–7.1)
	Pharmacy/laboratory	29	21	1	24	37	1
Is there enough staff	Yes	93	54	0.62(0.3–1.52)	76	84	1.5(0.67–3.19)
	No	56	18	1	25	29	1
Having clear responsibility	Yes	125	64	2.5(0.8–8.29)	85	99	0.87(0.32–2.3)
	No	24	8	1	16	14	1
equipment’s	Often	104	50	1.3(0.55–3.15)	81	94	0.91(0.38–2.2)
	sometimes	45	22	1	20	19	1
Injured while at work during last 12 months	Yes	8	3	0.77(0.2–4.9)	5	3	0.9(0.17–4.5)
	No	141	69	1	96	110	1
uncomfortable room temperature	Yes	121	31	**6.2(2.63-14.1) ∗**	87	73	**3.1(1.40-6.92) ∗**
	No	28	41	1	14	40	1

Moreover, in governmental facilities satisfied health professionals were 74 % less likely to be stressed than unsatisfied professionals (AOR = 0.26, 95%CI: 0.12–0.50).

In private facilities, health professionals who were getting enough support and assistance from other coworkers were found to be 78% less stressed (AOR = 0.22, 95%CI: 0.06, 0.83**) (**Table 9**).**

**Table 9:** Interpersonal relationship and career developmental factors and its association with work-related stress among health professionals by institutional category, zone 1 Afar region, Ethiopia 2021.

Interpersonal relationship and career developmental factors		Governmental			Private		
		Stress	No stress	Adjusted OR (95%CI)	stress	No stress	Adjusted OR (95%CI)
Do you feel getting enough support and assistance?	Yes	93	54	0.42(0.11–1.6)	76	84	**0.22(0.06-0.83) ∗**
	No	56	18	1	25	29	1
Receiving necessary trainings	Often	19	12	0.6(0.19–1.77)	12	20	0.42(0.17–1.07)
	Sometimes	32	32	0.25(0.1–1.01)	37	46	0.94(0.45–1.9)
	Rarely/not at all	98	28	1	52	47	1
Violence from patients	Yes	43	14	0.75(0.3–2.04)	31	24	1.3(0.57–2.9)
	No	106	58	1	70	89	1
currently involved in any conflict	Yes	14	3	11.3(0.82–28.9)	5	4	2.4(0.5–11.21)
	No	135	69	1	96	109	1
Having fair career development opportunities	Yes	99	58	0.49(0.2–1.18)	63	61	1.8(0.93–3.4)
	No	50	14	1	38	52	1
Job satisfaction	Satisfied	62	51	**0.26(0.12-0.5) ∗**	51	82	**0.32(0.16-0.64) ∗**
	Dissatisfied	88	20	1	50	31	1

## 4 Discussion

The study's main goal was to determine the level of work-related stress (WRS), compare it between government and private facilities, and find associated factors with WRS among healthcare employees in both private and governmental health facilities in Afar's zone 1.

Our study found that the magnitude of work-related stress among governmental institutions of health professionals was 67.5%, which is lower than a study report from Egypt (98.5 percent of health professionals from government health institutions showed moderate to severe stress levels). This might be due to the difference in objectives of the two studies (the Egyptian study was aimed at assessing the perceived stress level, which we believe that it is likely to result in a high level of perceived stress level. Moreover, the existence of differences in the working region might be the other possible explanation for this difference, which is supported by the Peruvian study [10, 11]. However, it was higher than the results reported from Mekelle, Ethiopia (46.9%). The possible explanation for this might be due to the occurrence of the COVID-19 global pandemic, which could increase health professionals' work-related stress levels in the current study area. Since health professional workers are the most vulnerable groups to this pandemic [12]. Furthermore, our study findings are higher than the study conducted in Saudi Arabia (55%). This might be due to due the difference in the study settings [13].

Moreover, in this study, the magnitude of work-related stress among private health institutions was 47.2%. Which is lower than health workers from governmental institutions. This might be due to the difference in the study settings and incentives. Health workers from private facilities are having a more suitable working environment, and working hours, which could be an explanation for the difference in the two setups.

Furthermore, the overall prevalence of work-related stress in this study was 57.5%, which was aligned with studies done in Britain [14]. However, it was higher than the results reported Nigeria (31.6%), and Addis Ababa(37.8%) [2, 15]. The possible reasons for this difference might be due to differences in the level of institutions (explained by the availability of more trained staff and availability of sufficient equipment and supplies in previous studies), and also differences in measuring tools and exposure to environmental hazards (explained by extreme hotness in the current study). The current study is lower than studies carried out in Ghana 69.5% [16], and Botswana 74% [16]. The possible reasons for the deviation might be due to organizational differences (explained by working hours, shortage of professionals, and increase in the number of patients in previous studies), study population (previous studies only included governmental facilities and some of them included a single professional group), and the relative difference in sample size in the current study.

This study also discovered that government healthcare workers in the Afar region are four times more likely to be stressed than those who work in private healthcare institutions. Inadequate staffing, a difference in the availability of equipment and drugs, a difference in monthly income, more patients/community violence in the government, and a lesser level of education among health care workers could all be contributing factors. Moreover, poor stress management and the difference in job satisfaction contributed to the difference in the WRS.

In this study, respondents' gender was a significant factor in stress in government facilities, male professionals were 38 percent less likely than females to report stress. This study supported the findings of Addis Ababa and Mekelle, which found that female health workers were more likely than males to experience stress [2, 12]. Similarly, the study conducted in Italy during COVID-19 pandemic shows, being female is more likely to be stressed than their counterparts [17]. This could be explained by females are more subject to community pressure and work-family interference (have multiple roles in family and social aspects). In addition, the monthly salary was found to be a major determinant of work-related stress in this study. Low-paid health professionals (4001–5000 ETB) were nearly three times more likely to be stressed than those who earned more than 7000 Birr per month. This is supported by a study conducted among nurses in Worabe specialized hospital in southwest, Ethiopia [18], and could be explained by lower paid professionals will have less ability to cover of their and their family's needs.

In this study, educational level and work experience had an insignificant association with occupational stress in the current study. However, it was contradicted by other studies done in Bahir Dar and Ghana [16, 19]. The possible explanation could be an organizational difference (This may be explained by different tasks and working conditions in different setting, giving up to exposure to different sources of stress) and a difference in measurement tool used in previous studies.

Workload, job satisfaction, and an uncomfortable room temperature were found to be the major sources of stress for health professionals in both government and commercial hospitals, according to the survey. In both public and private institutions, the workload was the most important predictor of work-related stress; as a result, professionals who worked 40 h per week were roughly half as likely to be stressed as those who worked more than 50 h per week. As a result, the likelihood of experiencing stress rises as the workload rises. This is consistent with other studies conducted in Addis Ababa and Bahirdar (Ethiopia), Saudi Arabia, and Nigeria [2, 7, 20, 21]; this can be explained by working for long hours, which causes fatigue and stress, as well as the high burden of the COVID-19 pandemic, a lack of staff, or inefficient human power allocations.

Furthermore, getting support and assistance in the workplace was a significant determinant of work stress among private facilities health professionals, accordingly, those who had enough support and assistance from other staff were 78% less likely to be stressed than those who didn’t have support and assistance. This could be due to the existence of a high workload and insufficient staff in private facilities and the exposure those health workers to long working hours [18, 20].

Moreover, job satisfaction was found to be an important factor for work-related stress among both groups of health professionals; the more satisfying health professionals with their jobs will be less stressed. The finding of this study complements the result of the survey done in Mekelle and Addis Ababa [12, 22]. The reason might be that dissatisfied workers have poor interaction and communication with staff and supervisors and low work moral decent. Furthermore, this result showed that health professionals who perceived the temperature as uncomfortable was found to be 4 times more likely to be stressed than those who didn’t complain about the temperature. This might be due to the uncomfortable working environment of the current study area. Since the Afar region is found in the east African refit valley and it is one of the lowest and hottest places in Ethiopia. The report from the International Labor Organization (ILO), which indicated that where exposure to hazardous agents like extreme temperature is a cause of workplace stress [22]. The possible reason for this association's existence is, that most of the time health professionals in developing countries including Ethiopia are forced to work for a long time without shift, and this practice of health workers along with the presence of uncomfortable room temperature results in the development of heat-related illness like lose consciousness, confusion, poor concentration and mental distress. Even this prone them to have work-related injury [23, 24].

### 4.1 Strength of the study

By using a comparative study method, this study tries to identify the difference in work-related stress and associated factors among health professionals of the two study groups.

### 4.2 Limitation of the study

The cross-sectional nature of this study was unable to show the cause-effect relationship of independent variables and dependent variables (work stress), and some of the measurements used to assess work-related stress may not be accurate due to subjective responses. Moreover, even if we are under the era of a COVID-19 pandemic, and this is more likely to affect the overall stress and anxiety level of healthcare professionals, our study did not show the association and impacts of COVID-19 pandemic on healthcare professional's work-related stress in the current study area.

## 5 Conclusion and recommendations

The prevalence of workplace stress was higher among health professionals both in governmental and private health facilities. Health professionals of governmental institutions were more exposed to work-related stress when compared to private institution professionals. The sex of the respondents and lack of support and assistance in the workplace were significant factors of work-related stress in governmental and private institutions respectively. While workload, job satisfaction, and uncomfortable room temperature were significant determinants of work-related stress in both governmental and private facilities.

Therefore, policymakers, the ministry of health, and the regional health bureau should set different strategies and programs focusing on stress reduction management programs in health facilities, improve the health management system through the application of a health sector transformational plan, allocate more funds to recruit health professionals as per standard to reduce workload, and to buy artificial air conditioner (AC) for the facilities to create a comfortable working environment, improving their salary and providing competency training programs to improve employee performance and update their professional knowledge and skills to reduce stress and coping with stress.

Motivational programs targeting female workers especially should have to be conducted routinely. Moreover, in a health facility setting, measures should be taken in create optimal organizational working hours, shifts, and rests based on the local situation, and establishing counseling centers with trained professionals to help health workers to cope with the stress.

## Declarations

### Author contribution statement

Sadat Mohammed Yesuf: Conceived and designed the experiments; Performed the experiments; Analyzed and interpreted the data; Contributed reagents, materials, analysis tools or data; Wrote the paper.

Behailu Tariku Derseh: Conceived and designed the experiments; Analyzed and interpreted the data; Contributed reagents, materials, analysis tools or data; Wrote the paper.

Daniel Girma: Conceived and designed the experiments; Contributed reagents, materials, analysis tools or data; Wrote the paper.

Tadesse Mamo Dejene: Conceived and designed the experiments; Performed the experiments; Analyzed and interpreted the data; Wrote the paper.

### Funding statement

This research did not receive any specific grant from funding agencies in the public, commercial, or not-for-profit sectors.

### Data availability statement

Data will be made available on request.

### Declaration of interest’s statement

The authors declare no conflict of interest.

### Additional information

No additional information is available for this paper.
